# Enhanced voltage regulation and current sharing of radial DC microgrids

**DOI:** 10.1038/s41598-026-62054-9

**Published:** 2026-07-20

**Authors:** Issra A. Mowafy, Ahmed H. EL-Ebiary, Mohamed Attia, Almoataz Y. Abdelaziz

**Affiliations:** 1 Electrical Power & Machines Department, Faculty of Engineering, Ain Shams University, Cairo, Egypt; 2 Electrical Power & Machines Department, Higher Institute of Engineering, El-Shorouk Academy, Cairo, Egypt; 3 Faculty of Engineering and Technology, Future University in Egypt, Cairo, 11835 Egypt

**Keywords:** Current sharing, DC microgrid, Voltage regulation, Energy science and technology, Engineering

## Abstract

DC microgrids are gaining more attention as their control is simpler, their efficiency is higher and they are more reliable. DC microgrid control aims to regulate load voltage at the nominal value and share load among distributed generation units with a certain ratio. This paper presents a control strategy for radial DC microgrids that achieves these two goals accurately and simultaneously. It doesn’t require communication among distributed generators. Only global sensing is required and only one signal is transmitted among DGUs. Tianji Horse Racing Optimization algorithm was used to tune controller parameters. A stability analysis was conducted to evaluate the effect of load change on system stability. This control method has much faster dynamic response compared to previously reported methods. MATLAB Simulink was used to build a model for the microgrid to test the proposed control strategy. Its stability was tested by applying sudden load changes. Also, plug-in and plug-out capability was verified. Constant power load was also used to test the proposed control strategy performance. Communication delay effect was also tested.

## Introduction

Distributed generation units (DGUs) including renewable energy-based generators, small conventional generators and energy storage systems are widely used in electrical power systems to increase reliability, reduce transmission losses and power cost. A microgrid is a combination of DGUs and loads that can be connected to the grid or islanded from it. According to buses voltages, a microgrid may be DC, AC or hybrid. DC microgrid reduces transmission losses as there is no skin effect that increases transmission line resistance in AC systems. DC microgrid also reduces conversion losses as many DGUs generate DC voltage and many loads need DC voltage such as speed-controlled motors, electric vehicles and LED lighting. It also has simpler control as there is no frequency or reactive power control^1^.

DC microgrid has many topologies such as radial, meshed, multi-bus, multi-terminal, zonal and ladder topologies. Radial DC microgrid is used for residential buildings. To control load sharing among dispatchable DGUs in a radial DC microgrid, droop control is used in the primary control level, but it has two disadvantages: current sharing accuracy is affected by line resistances, and it causes voltage drop at the load bus^2^. Nonlinear droop control is used to improve voltage regulation at light loads by using small droop gains and improve current sharing at heavy loads by using large droop gains^3–6^.

With linear droop control, secondary control was used to overcome its two problems. Secondary control may be centralized or distributed. Centralized control depends on a single controller for the whole microgrid that needs to communicate with all DGUs. This reduces reliability and scalability. In distributed control, each DGU has a controller and low-bandwidth communication channels with neighboring DGUs only. It depends on consensus control. So, it is suitable for large microgrids^2,7,8^.

Many researchers have focused on distributed control for the last years. In Han et al.^9^, one control loop was used in the zero-control level and reverse droop control was employed in the primary control layer to achieve fast dynamic response. In the secondary control level, a containment-based controller was used to regulate load bus voltage, and a consensus-based controller was used to improve current sharing among DGUs. In Dam and Lee^10^, two control loops were used in the zero-control layer and, power droop control was used at the primary control level. In the secondary control level, two PI controllers were used to add a shifted voltage to the primary control reference voltage to compensate for the bus voltage drop and correct the error in power sharing due to lines resistances.

In Guo et al.^11^, a pinning control was used by sending the load bus voltage to only one DGU to simplify the control method. In Liu et al.^12^, piecewise droop control was used to improve load sharing and voltage restoration. Droop curves are flexibly formed according to separated load regions. In Aryan et al.^13^, average current and Voltage sharing between DGUs was performed. In Metihalli and Sabhahit^14^, disturbance observer technique was used to estimate disturbances, then distributed consensus control was used to suppress them. It also controls current sharing among batteries in case of external disturbance.

In Xing et al.^15^, virtual voltage drop (voltage drop due to droop control and line resistance) was utilized in the secondary control phase. In Xing et al.^16^, varying droop gains were used to allow adjustable current sharing ratio. In Trinh and Chung^17^, a controller was used to minimize operation cost and regulate the load bus voltage by scheduling charging and discharging electric vehicles. In Nishita et al.^18^, a distributed control was used to control batteries charge and discharge to maximize the duration when the microgrid operates independently.

In Zhang et al.^19^, distributed droop-free control was used. A discrete dynamic consensus algorithm was used to estimate a virtual state variable including voltage and current information. In Aluko et al.^20^, secondary controller parameters were optimized using a new flight-based artificial bee colony algorithm. In Onaolapo et al.^21^, fuzzy logic control was accustomed to adapting the integral control constant. In Xue et al.^22^, incremental cost droop was used to achieve economic power sharing and dynamic average consensus control was utilized to regulate the DC bus voltage.

In Guo et al.^23^, event triggered data sampling and transmitting was used to decrease communication cost. In Chai et al.^24^, fixed time control was employed at the secondary control level. In Wang et al.^25^, virtual voltage compensation served as the secondary controller. In Zhang et al.^26^, control was designed to optimize power flow in the microgrid. Power flow constraint is quadratic, so a convex relaxation strategy was applied to linearize this constraint. In Wang et al.^27^, the convex relaxation method was used with a microgrid with a constant power load.

In Xing et al.^28^**,** virtual voltage drop was divided by line inductance to give virtual current derivative. In Nguyen and Pham^29^, delta iteration method was used in the secondary control level. In Jabbar et al.^30^, an on-grid microgrid with a wind power unit, electric vehicle and battery unit was used. Control system was designed to extract maximum power from wind and PV units, minimize the cost of energy absorbed from the grid and regulate the state of charge of the battery and electric vehicle. In Chen et al.^31^, adaptive event-triggered control was employed to reduce information transmission losses. In Zaery et al.^32^, arbitrary time control was used for bus voltage regulation to achieve a user-defined settling time and improve economic operation.

In previous research, to achieve current sharing among DGUs and regulate load bus voltage, DGUs needed to share their voltages and currents or secondary control signals with each other. This caused communication delays that increase with the number of DGUs in the microgrid due to network congestion. Proposed control strategy transmits only one signal (load bus voltage) to DGUs. It also has short execution time as it uses only zero level control. Also, different communication delays among DGUs don’t affect current sharing among them. So, there is no synchronization delay. Some parameters of the system were modified to improve its dynamic performance. Tianji Horse Racing Optimization algorithm was used to tune PI controller parameters to improve system dynamics. The control strategy was tested using resistive load and constant power load. Also, plug in and plug out capability and effect of time delay were tested.

## DC microgrid structure

**Fig. 1:**
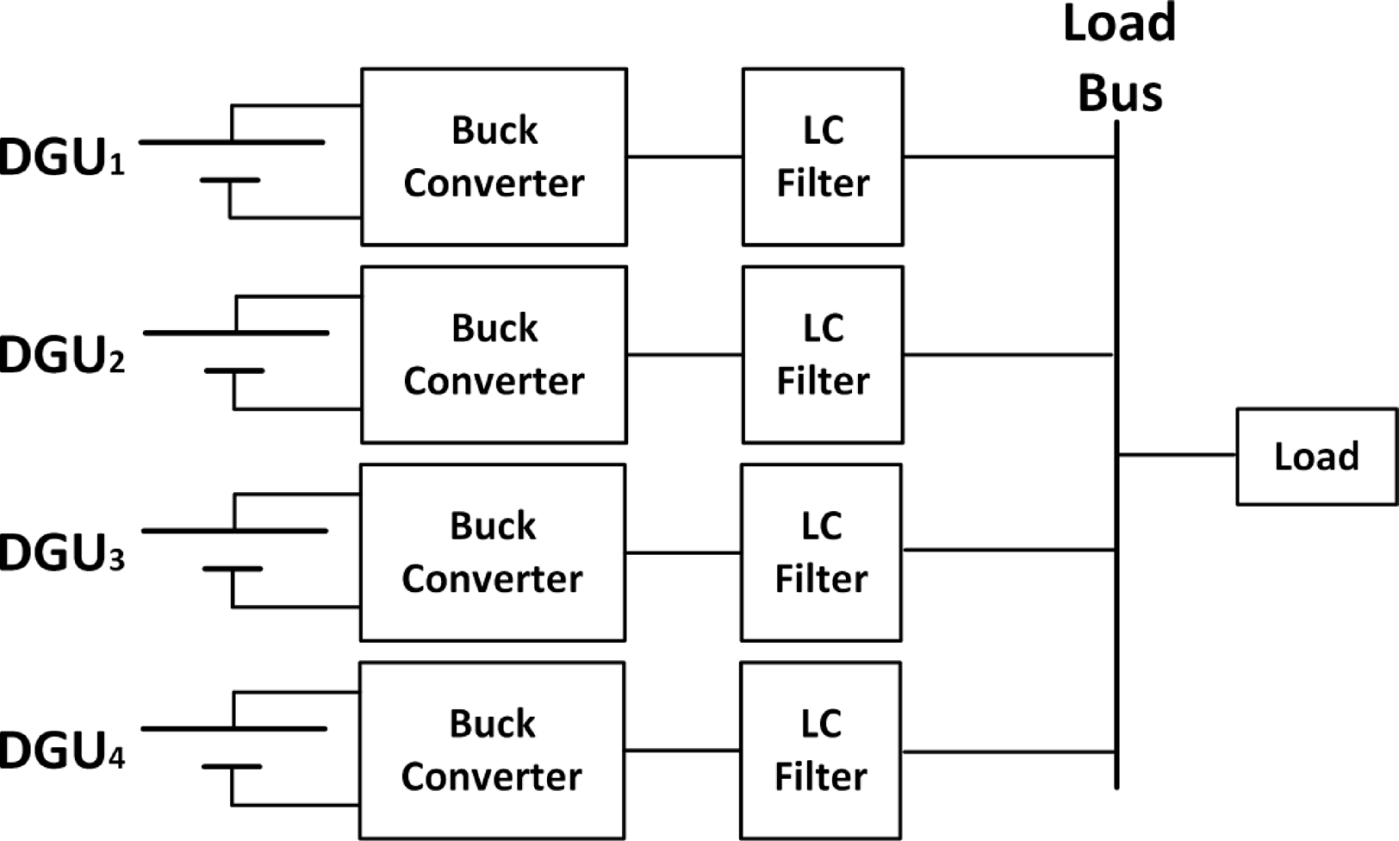
Radial DC microgrid structure.

In a radial DC microgrid, all loads are connected to a single bus and DGUs are connected to it through lines as shown in Fig. 1.

So,

1 $$V_{b} = V_{i} - r_{i} I_{i} , \quad i = 1,2, \ldots ,N$$

where ${V}_{b}$ is the load bus voltage, ${V}_{i}$ and ${I}_{i}$ are the output voltage and current of the ${i}^{th}$ DGU respectively, ${r}_{i}$ is the resistance of the line connecting the ${i}^{th}$ DGU to the load bus, and $N$ is the number of DGUs in the microgrid.

DGUs are interfaced by buck converters. Zero level control of buck converters includes two control loops: voltage control loop and current control loop. So,

2 $${I}_{{Lref}_{i}}={k}_{pv}{e}_{{v}_{i}}+\int {k}_{iv}{e}_{{v}_{i}}dt$$

where ${I}_{{Lref}_{i}}$ is the reference inductor current of the ${i}^{th}$ converter, ${k}_{pv}$ and ${k}_{iv}$ are the proportional and integral gains of the PI controller used in voltage control loop respectively, and

3 $${e}_{{v}_{i}}={V}_{{ref}_{i}}-{V}_{i}$$

where ${V}_{{ref}_{i}}$ is the reference voltage for zero level control of the ${i}^{th}$ converter.

Also,

4 $${D}_{i}={(k}_{pi}{e}_{{i}_{i}}+\int {k}_{ii}{e}_{{i}_{i}}dt)/100$$

where ${D}_{i}$ is the ${i}^{th}$ converter duty cycle, ${k}_{pi}$ and ${k}_{ii}$ are the proportional and integral gains of the PI controller used in current control loop respectively, and

5 $${e}_{{i}_{i}}={I}_{{Lref}_{i}}-{I}_{{L}_{i}}$$

where ${I}_{{L}_{i}}$ is the ${i}^{th}$ converter inductor current.

## DC microgrid proposed control method

It’s required that DGUs share load current in a direct proportion to each DGU rating such that

6 $${k}_{i}{I}_{i}={k}_{j}{I}_{j} ,\quad \forall i,j = \mathrm{1,2},\dots ,N$$

where ${k}_{i}$ is a constant inversely proportional to the ${i}^{th}$ DGU rating. It’s also required to regulate the load bus voltage (${V}_{b}$*)* at the nominal value ${V}^{*}$

7 $${V}_{b}={V}^{*}$$

**Fig. 2:**
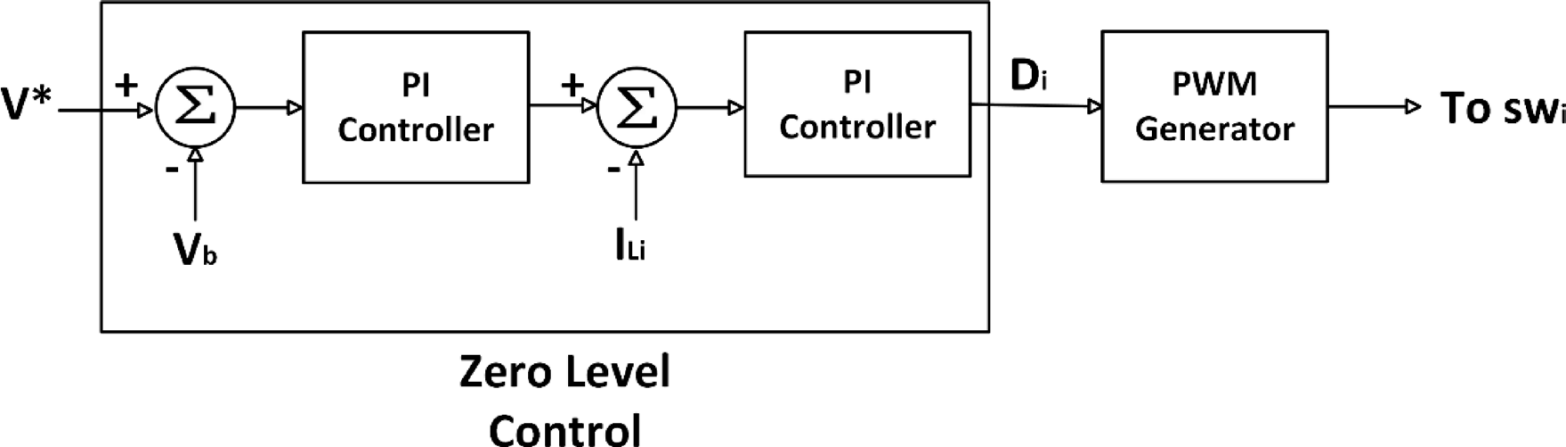
Proposed control strategy.

These two objectives are accurately achieved using the proposed control method shown in Figure 2.

To regulate the load bus voltage at its nominal value, ${e}_{{v}_{i}}$ is calculated as

8 $${e}_{{v}_{i}}={V}^{*}-{V}_{b}$$

More than one sensor should be used to measure ${V}_{b}$ to increase system reliability. As the voltage control loop calculates the inductor current reference, PI controllers’ parameters in the voltage control loop are designed to be proportional to the DG unit rating. So,

9 $$\begin{aligned} & k_{i} {k}_{{pv}_{i}} = k_{j} k_{{pv}_{j}} , \\ & {\mathrm{and}}\;\;k_{i} k_{{iv}_i} = k_{j} k_{{iv}_j} , \quad i,j = 1,2, \ldots ,N \\ \end{aligned}$$

A software limiter is used so that ${I}_{{Lref}_{i}}$ can’t exceed the rated current of each DGU for a period exceeding system settling time. To prevent negative output currents during starting, improve dynamic response and current sharing among DGUs and reduce current ripples, converters capacitances are designed to be directly proportional to their rating. So,

10 $$k_{i} C_{i} = k_{j} C_{j} ,\quad i,j = 1,2, \ldots ,N$$

where ${C}_{i}$ is the ${i}^{th}$ converter capacitance. Inductance and capacitance of a buck DC-DC converter are inversely proportional to each other for limited current and voltage ripples^33^. So, converters inductances are set to be inversely proportional to their rating.

11 $$k_{i} L_{j} = k_{j} L_{i} , \quad \forall i,j = 1,2, \ldots ,N$$

where ${L}_{i}$ is the ${i}^{th}$ converter inductance.

For a fast dynamic response for current control loops, their PI controllers’ parameters are set to be directly proportional to converters inductances. So,

12 $$\begin{aligned} & k_{i} k_{{pi_{j} }} = k_{j} k_{{pi_{i} }} , \\ & {\mathrm{and}}\;\;~k_{i} k_{{ii_{j} }} = k_{j} k_{{ii_{i} }} ,\;\;i,j~ = ~1,2, \ldots ,N \\ \end{aligned}$$

To design PI controllers’ parameters of voltage control loops, Tianji Horse Racing Optimization (THRO) algorithm was used. It has better convergence rate and solution precision due to the unique dynamic individual matching strategy used^34^. Next section explains it briefly.

## Tianji Horse racing optimization (THRO) algorithm

This algorithm is inspired by a story that happened in China over 2000 years ago. There was horse racing between the Qi state prominent military leader called Tianji and the King. Each racer had three horses: slow, medium and fast. Tianji’s military advisor, Sunbin, advised him to challenge King’s fast horse with his slow horse, King’s medium horse with his fast horse and King’s slow horse with his medium horse to lose the first round only and win the other two rounds.

**Fig. 3:**
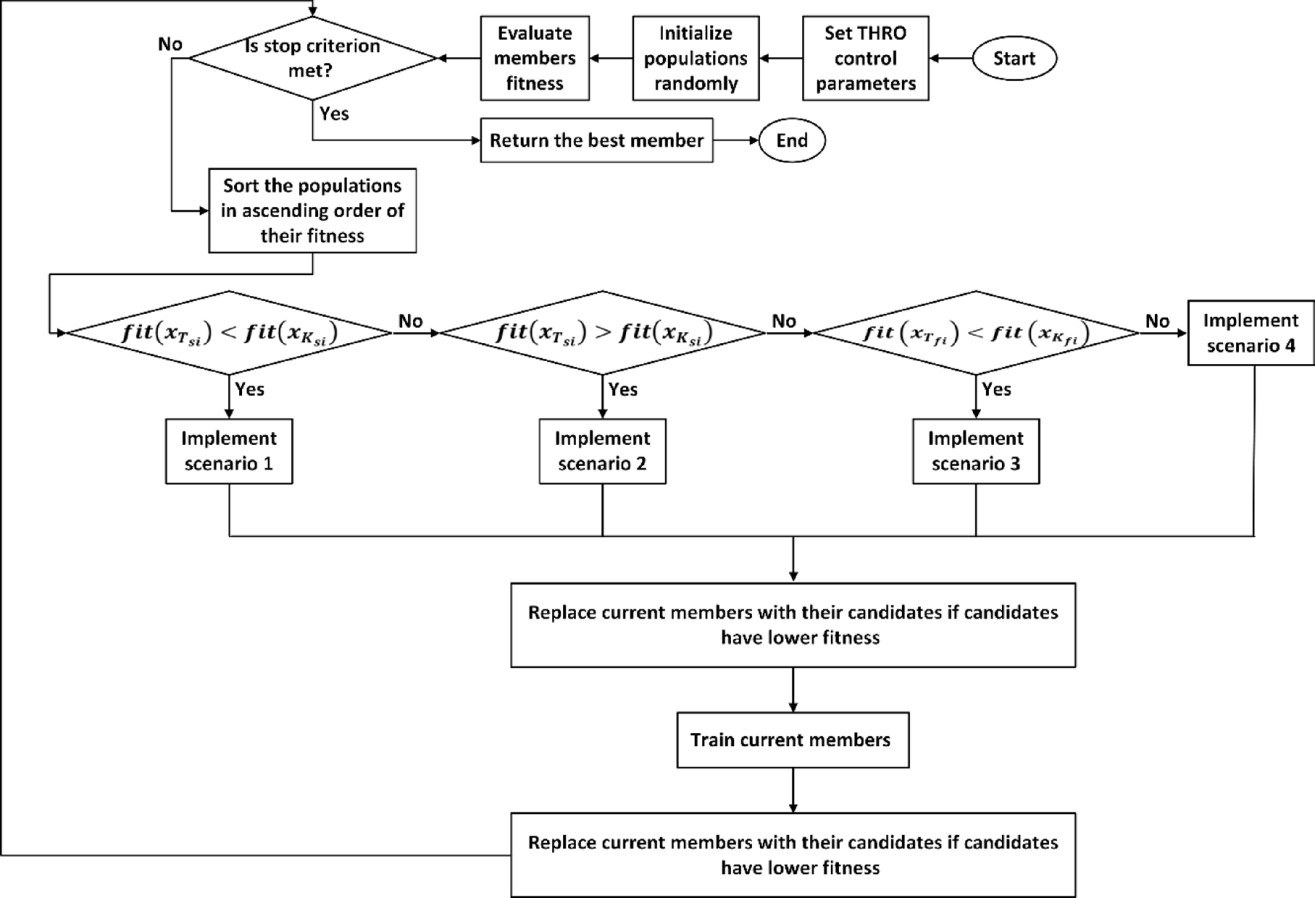
THRO flowchart.

In the algorithm, the total population is divided into two populations, Tianji’s horses’ population and King’s horses’ population. Each population has $n$ horses. So, there are $n$ rounds in each iteration. After each round the two competitor horses are removed from the two populations. For a minimization problem, each population is ranked in ascending order of the fitness value. Then, one of four scenarios is applied.

1. If Tianji’s current last member has a lower fitness value than King’s, they compete and Tianji wins. Tianji’s current last member will be updated with respect to Tianji’s best member, and King’s current last member will be updated in relation to Tianji’s current last member.
2. If Tianji’s current last member has a larger fitness value than King’s, it competes with King’s current first member. Tianji’s current last member is updated with respect to a random member in its population, and King’s current first member is updated in relation to the best member in King’s population totally.
3. If the current last members in both populations have the same fitness value and Tianji’s current first member has a smaller fitness value than King’s, the current first members of both populations compete. They are refreshed with respect to the best member in Tianji’s population totally.
4. If the fitness value of the current last members in both populations is the same, and Tianji’s current first member fitness value is larger than or equal to the King’s, Tianji’s current last member competes with the King’s current first member.

After $n$ rounds, populations members are trained, to prevent stagnation during solutions reinforcement, using randomly chosen members to improve their fitness value gradually or using the best member in the population to reach their full potential. The following algorithm presents the Pseudo-code of THRO, and its flowchart is shown in Fig. 3.

**Algorithm 1:**
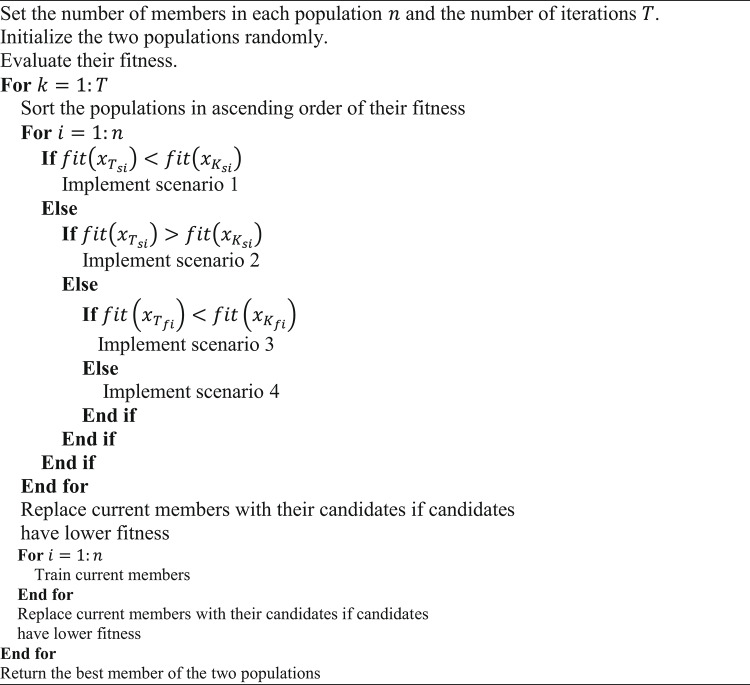
Pseudo-code of THRO

## System parameters design

The DC microgrid shown in Fig. 1 was used to verify the proposed control method performance. Its parameters are shown in Table 1. Converter capacitances values were set so that ${\sum}_{i=1}^{4}{C}_{i}=0.0088$, and converter inductances values were set so that ${\sum}_{i=1}^{4}{L}_{i}=0.04$ (the same value as models used in Ref^15^ and^16^). The current loop controller parameters were set so that ${\sum}_{j=1}^{4}{{k}_{pi}}_{j}=20$ and ${\sum}_{j=1}^{4}{{k}_{ii}}_{j}=440$

**Table 1:** System parameters.

	DGU 1	DGU 2	DGU 3	DGU 4
${k}_{pv}$	$10.7635$	$21.527$	$32.2905$	$32.2905$
${k}_{iv}$	$796.1536$	$1592.3072$	$2388.4608$	$2388.4608$
${k}_{pi}$	$9.2308$	$4.6154$	$3.0769$	$3.0769$
${k}_{ii}$	$203.0769$	$101.5385$	$67.6923$	$67.6923$
$C$	$0.00098 \,\mathrm{F}$	$0.00196\,\,\mathrm{F}$	$0.00294 \,\mathrm{F}$	$0.00294 \,\mathrm{F}$
$L$	$0.0186 \,\mathrm{H}$	$0.0093\, \mathrm{H}$	$0.0062 \,\mathrm{H}$	$0.0062 \,\mathrm{H}$
$r$	$0.2\, \Omega$	$0.1\,\Omega$	$0.2\, \Omega$	$0.3\, \Omega$
$k$	$6$	$3$	$2$	$2$
${V}_{DC}$	$100 \,\mathrm{V}$	$100 \,\mathrm{V}$	$100 \,\mathrm{V}$	$100 \,\mathrm{V}$
${f}_{s}$	$1.25 \,\mathrm{k}\mathrm{H}\mathrm{z}$	$1.25\,\mathrm{k}\mathrm{H}\mathrm{z}$	$1.25\, \mathrm{k}\mathrm{H}\mathrm{z}$	$1.25 \,\mathrm{k}\mathrm{H}\mathrm{z}$
${V}^{*}$	$48 \,\mathrm{V}$	$48\, \mathrm{V}$	$48 \,\mathrm{V}$	$48 \,\mathrm{V}$

To set the values of voltage loop controller parameters, THRO algorithm was used with a model for the microgrid built in MATLAB Simulink. The solver type was Fixed-step (ode3) with step size:1e−7. Initially, a resistive load of $5\Omega$ was connected. At $t=0.4 \mathrm{s}$, another resistive load of $5\Omega$ was connected in parallel with the first load. At $t=0.7 \mathrm{s}$, the second load was disconnected. The objective function ($F$) was calculated as

13 $$F={\int}_{0}^{1}{({V}^{*}-{V}_{b})}^{2}dt$$

Subjected to:

14 $$\begin{aligned} k_{{{pv}_{2} }} & = 2k_{{{pv}_{1}}} ,~~~k_{{iv}_{2} } = 2k_{{iv}_{1} } \\ k_{{pv}_{3} } & = k_{{pv}_{4}} = 3k_{{pv}_{1}} ,~~~k_{{iv}_{3}} = k_{{iv}_{4}} \\ & = 3k_{{iv}_{1}} \\ 0 \le& {k}_{{pv}_1}\le 30, \,\, 0\le{k}_{{iv}_1}\le1500 \end{aligned}$$

The decision variables were ${k}_{{pv}_{1}}$ and ${k}_{{iv}_{1}}.$ Maximum iteration was set as $20$, and population size was set as $20$. The best solution was ${k}_{{pv}_{1}}=10.7635, {k}_{{iv}_{1}}=796.1536$

## Stability and sensitivity analyses

Using small signal model of buck converters presented in^33^,

15 $${G}_{{V}_{i}}=\frac{\Delta {V}_{i}}{\Delta {D}_{i}}=\frac{{V}_{DC}}{{L}_{i}{C}_{i}{s}^{2}+\frac{{L}_{i}}{{R}_{i}}s+1}$$

16 $$G_{{I_{i} }} = \frac{{{{\Delta }}I_{{L_{i} }} }}{{{{\Delta }}D_{i} }} = \frac{{V_{{DC}} \left( {C_{i} s + \frac{1}{{R_{i} }}} \right)}}{{L_{i} C_{i} s^{2} + \frac{{L_{i} }}{{R_{i} }}s + 1}}$$

where ${V}_{DC}$ is the converters input voltage and ${R}_{i}$ is the resistance connected to each converter. After neglecting line resistances ${R}_{i}$ is calculated as

17 $${R}_{i}={R}_{L}{k}_{i}* \sum\limits_{j=1}^{N}{{k}_{j}}^{-1}$$

For current control loop, the overall transfer function (${T}_{{I}_{i}}$) is calculated as

18 $${T}_{{I}_{i}}=\frac{\Delta {D}_{i}}{\Delta {{I}_{Lref}}_{i}}=\frac{0.01({{k}_{pi}}_{i}+\frac{{{k}_{ii}}_{i}}{s})}{1+{0.01({{k}_{pi}}_{i}+\frac{{{k}_{ii}}_{i}}{s})G}_{{I}_{i}}}$$

For voltage control loop, the overall transfer function (${T}_{{V}_{i}}$) is calculated as

19 $$T_{{V_{i} }} = \frac{{{{\Delta }}D_{i} }}{{{{\Delta }}V_{{ref_{i} }} }} = \frac{{\left( {k_{{pv_{i} }} + \frac{{k_{{iv_{i} }} }}{s}} \right)T_{{I_{i} }} }}{{1 + \left( {k_{{pv_{i} }} + \frac{{k_{{iv_{i} }} }}{s}} \right)T_{{I_{i} }} G_{{V_{i} }} }}$$

Then the system characteristic equation is

20 $$\begin{aligned} & a_{0} + a_{1} s + a_{2} s^{2} + a_{3} s^{3} + a_{4} s^{4} = 0 \\ & a_{0} = 0.01V_{{DC}} k_{{iv_{i} }} k_{{ii_{{_{i} }} }} \\ & a_{1} = 0.01V_{{DC}} \left( {\frac{{k_{{ii}_{i}} }}{{R_{i} }} + k_{{pv_{i} }} k_{{ii_{{_{i} }} }} + k_{{iv_{i} }} k_{{pi_{i} }} } \right) \\ & a_{2} = 1 + 0.01V_{{DC}} \left( {\frac{{k_{{pi_{i} }} }}{{R_{i} }} + k_{{ii_{i} }} C_{i} + k_{{pv_{i} }} k_{{pi_{i} }} } \right) \\ & a_{3} = \frac{{L_{i} }}{{R_{i} }} + 0.01V_{{DC}} k_{{pi_{i} }} C_{i} \\ & a_{4} = L_{i} C_{i} \\ \end{aligned}$$

Using system parameters shown in Table 1 and Eq. 17, the characteristic equation for all DGUs becomes

21 $$\begin{aligned} & 161680.405 + \left( {9534.953 + \frac{22.564}{{R_{L} }}} \right)s + \left( {100.555 + \frac{1.026}{{R_{L} }}} \right)s^{2} \\ & \quad + \left( {9.046 \times 10^{ - 3} + \frac{{2.067 \times 10^{ - 3} }}{{R_{L} }}} \right)s^{3} + 1.823 \times 10^{ - 5} s^{4} = 0 \\ \end{aligned}$$

**Fig. 4:**
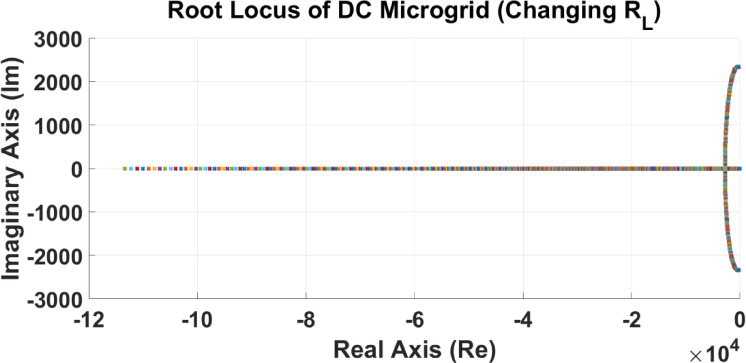
System poles root locus as ${R}_{L}$ varies.

The root locus of poles as ${R}_{L}$ varies is shown in Fig. 4. All poles are in the left-hand side of the s-plane, so the system is stable. Under CPL, the source output impedance (${Z}_{o}$) is zero, and Middlebrook criterion $\left|\frac{{Z}_{o}}{{Z}_{i}}\right|<1$ is always satisfied. So, the system is always stable.

**Fig. 5:**
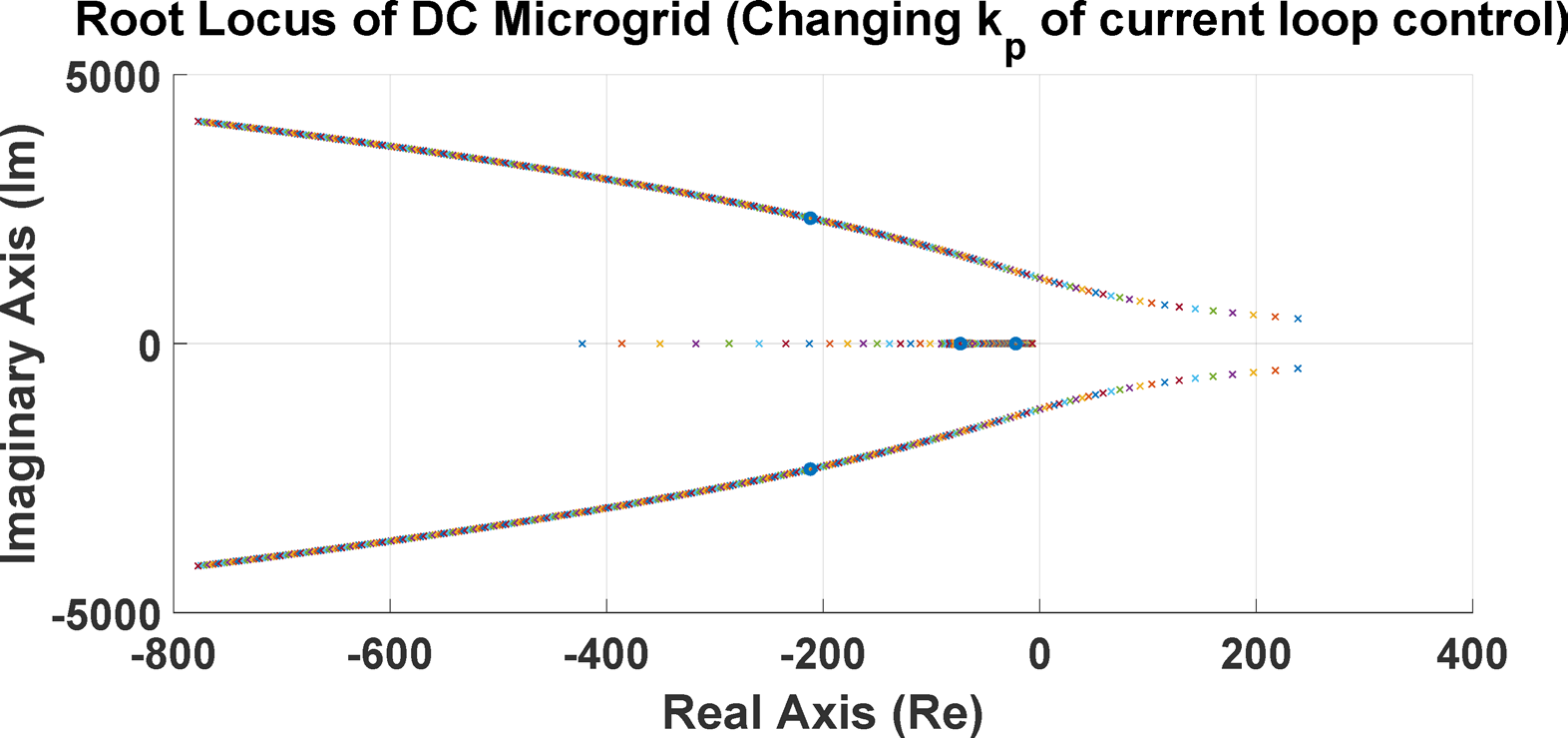
System poles root locus as ${k}_{{pi}_{i}}$ varies.

**Fig. 6:**
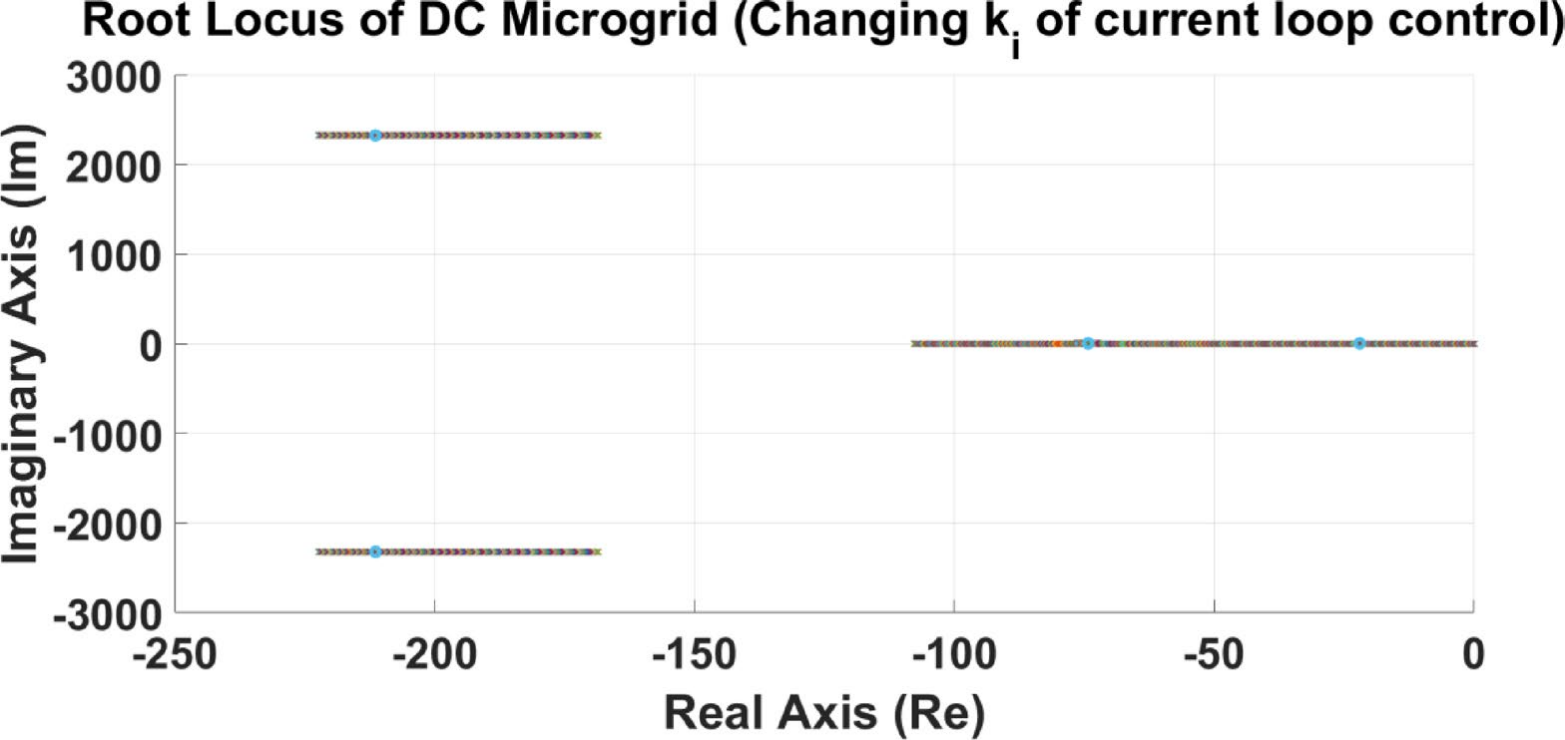
System poles root locus as ${\mathrm{k}}_{\mathrm{ii}_\mathrm{i}}$ varies.

Sensitivity analysis was also conducted. The root locus of poles as ${k}_{{pi}_{1}}$ varies is shown in Fig. 5. Poles at ${k}_{{pi}_{1}}=9.2308$ are represented by ($o$) symbol. Also, the root locus of poles as ${k}_{{ii}_{1}}$ varies is shown in Fig. 6. Poles at ${k}_{{ii}_{1}}=203.0769$ are represented by ($o$) symbol. Chosen values of current loop control gains guarantee good dynamic performance and stable operation.

## Plug-in control

For a DC microgrid with $N$ operating DGUs, to connect an additional DGU ($m$), its inductor current reference is calculated as

22 $${I}_{{Lref}_{m}}={I}_{Load}/(1+{k}_{m}\sum_{i=1}^{N}{{k}_{i}}^{-1})$$

where ${I}_{Load}$ is the total load current.

After the additional DGU reaches steady state, the inductor current reference is calculated as

23 $${I}_{{Lref}_{m}}={I}_{{m}_{ss}}+{k}_{{pv}_{m}}{e}_{{v}_{m}}+\int {k}_{{iv}_{m}}{e}_{{v}_{m}}dt$$

where ${I}_{{m}_{ss}}$ is the steady-state value of ${I}_{m}$.

## Simulation and results

The proposed control strategy was tested during sudden load changes, its plug-in and plug-out capability, its performance with constant power loads and communication delay effect using the microgrid shown in Figure 1 with parameters shown in Table 1. Results are shown in this section

1. Sudden load change test

In this case study, load was suddenly doubled or halved. The four DGUs supplied the load. For $t=0 :2 \mathrm{s}$, a resistive load of $5\Omega$ was connected. At $t=2 \mathrm{s}$, another resistive load of $5\Omega$ was connected in parallel with the first load. At $t=4 \mathrm{s}$, the second load was disconnected.

**Fig. 7:**
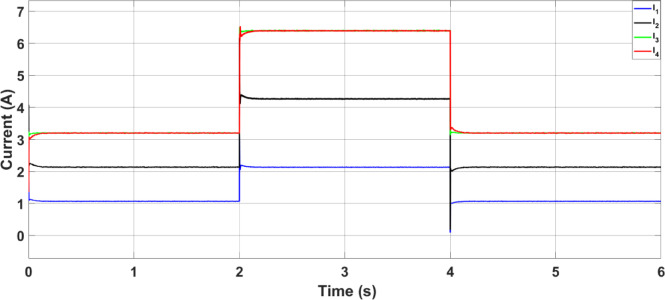
Output currents for sudden load changes test.

**Fig. 8:**
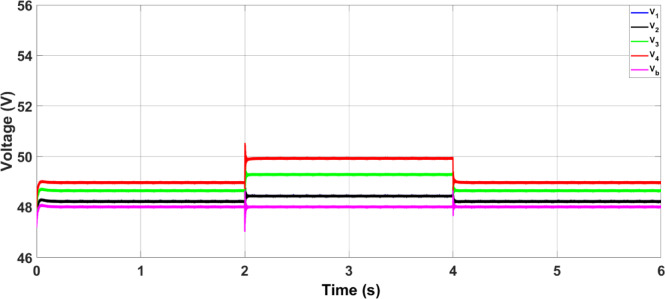
Voltages for sudden load changes test.

Output currents of the four DGUs are shown in Fig. 7, and output voltages and load voltage are shown in Fig. 8. Current sharing became more accurate. Line resistances did not affect it. To evaluate current sharing accuracy, current sharing error (${err}_{cs}$) is calculated as

24 $$\begin{aligned} & err_{cs} = \mathop \int \limits_{0}^{6} \left(\mathop \sum \limits_{i = 1}^{4} err_{i} \right)dt \\ & err_{i} = \left( {I_{i}^{*} - I_{i} } \right)^{2} \\ & I_{i}^{*} = I_{Load} *d_{i}^{ - 1} /\left( {\mathop \sum \limits_{j = 1}^{4} d_{j}^{ - 1} } \right) \\ \end{aligned}$$

${err}_{cs}$ value decreased by $99.21\%$ (from $0.6552$ to $0.0052$) in comparison with case study 1 in^16^. Current maximum peak overshoot is shown in Table 2. As it lasts for a very short duration ($0.01 s$), its effect is negligible.

**Table 2:** Current maximum peak overshoot ($MPOS\%$).

DGU1	DGU2	DGU3	DGU4
44.77	44.92	1.44	3.45

Time response parameters for load bus voltage such as maximum peak overshoot (*MPOS%*) and settling time (*T*_*settling*_) are shown in Table 3. A comparison with case study 1 in^16^ is shown in Table 4. Maximum undershoot (MUS) reduction percentage can be calculated as

25 $$\frac{1.06-0.99}{1.06}\times 100\%=6.6\%$$

and settling time reduction percentage can be calculated as

**Table 3:** Time response specifications.

Case Study	MPOS%	T_settling_
Sudden load change test	$1.3758$	$0.05$
Plug-in and plug-out test	$0.5781$	$0.05$
Constant power load test	$0.6838$	$0.05$

**Table 4:** Comparison with case study 1 in^16^.

Case Study	MUS (V)	Settling time (s)
Case study 1 in^16^	$1.06$	$0.4$
Current case study	$0.99$	$0.05$

26 $$\frac{0.4-0.05}{0.4}\times 100\%=87.5\%$$

(2) Plug-in and plug-out capability test

In this case study, a constant load of $5\Omega$ was connected, and one DGU was disconnected and connected again to evaluate the proposed control strategy performance. For $t=0 :2 \mathrm{s}$, the four DGUs operated. At $t=2 \mathrm{s}$, DGU4 was disconnected. At $t=4 \mathrm{s}$, DGU4 was connected again.

**Fig. 9:**
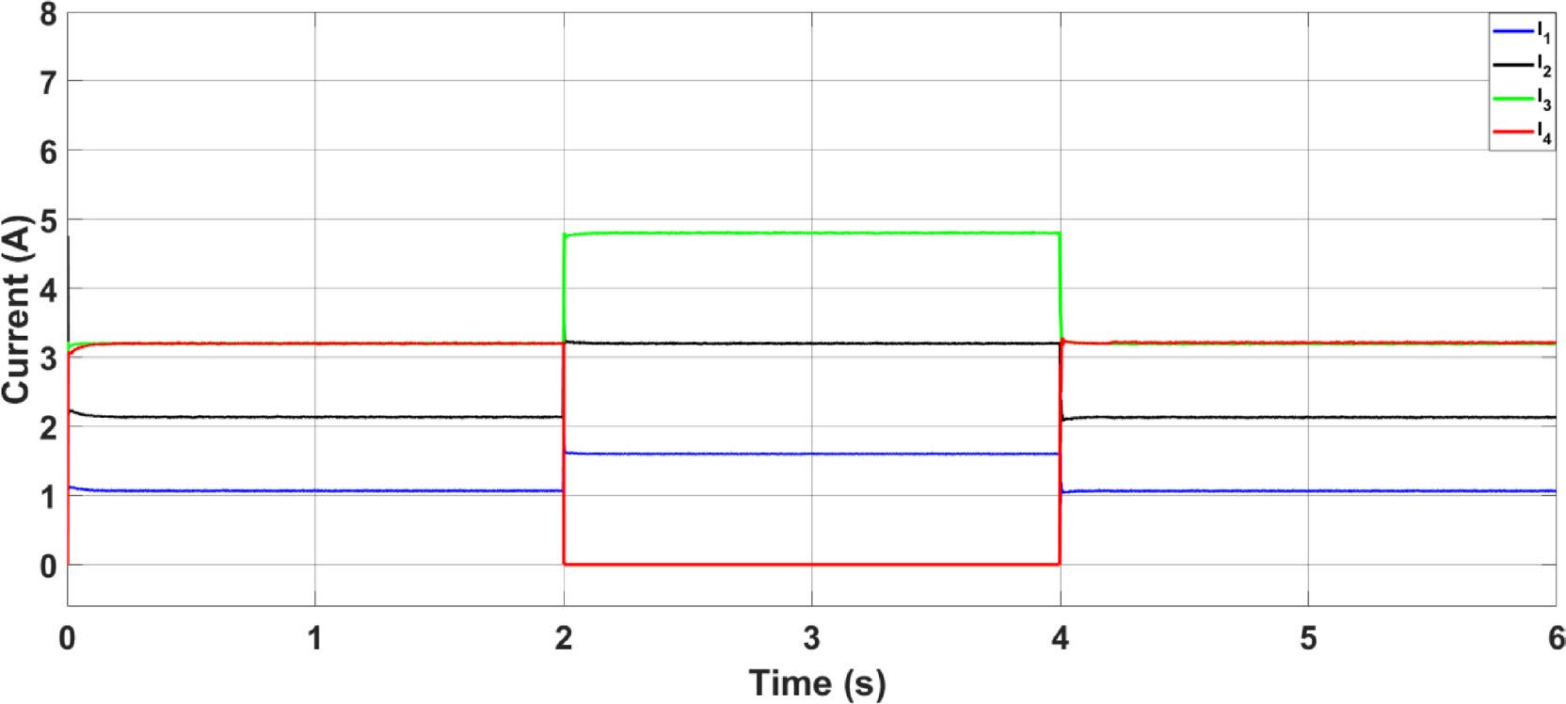
Output currents for plug-in and plug-out capability test.

**Fig. 10:**
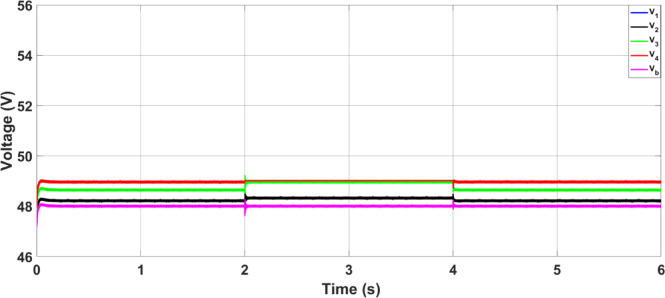
Voltages for plug-in and plug-out capability test.

DGUs’ output currents are shown in Fig. 9. Output voltages and load bus voltage are shown in Fig. 10. Load current was accurately shared among remaining DGUs after DGU4 disconnection and reconnection. Compared to case study 3 in^15^, maximum undershoot for load voltage was reduced by $51.4\%$ (from $0.823 \mathrm{V}$ to $0.4 \mathrm{V}$).

(3) Constant power load test

Constant power load has a negative incremental impedance. A decrease in voltage leads to an increase in current. So, it decreases DC microgrid stability. It was used to test the proposed control strategy stability. For $t=0 :2 \mathrm{s}$, a resistive load of $5\Omega$ was connected in parallel with a constant power load of $200 \mathrm{w}$. At $t=2 \mathrm{s}$, another constant power load of $200 \mathrm{w}$ was connected in parallel. At $t=4 \mathrm{s}$, a constant power load of $200 \mathrm{w}$ was disconnected.

**Fig. 11:**
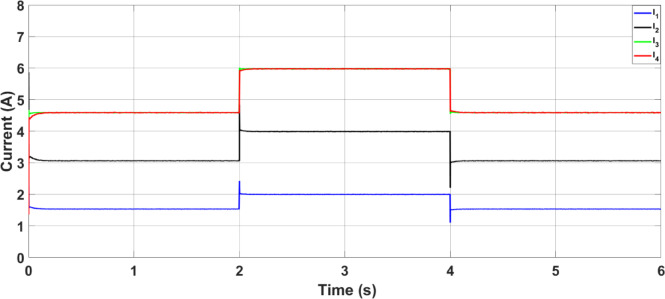
Output currents for constant power load test.

**Fig. 12:**
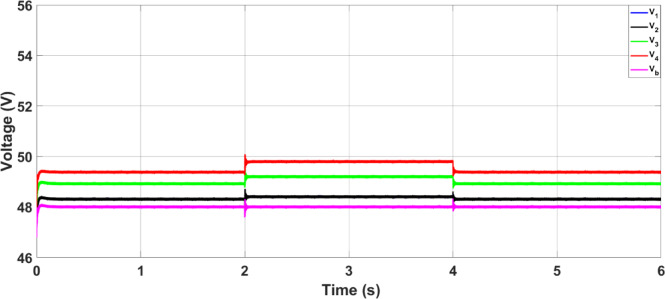
Voltages for constant power load test.

Results are shown in Figs. 11 and 12. Compared to case study 1 in^28^, maximum undershoot for load voltage was reduced by $83.85\%$ (from $2.6 \mathrm{V}$ to $0.42 \mathrm{V}$).

(4) Communication delay test

**Fig. 13:**
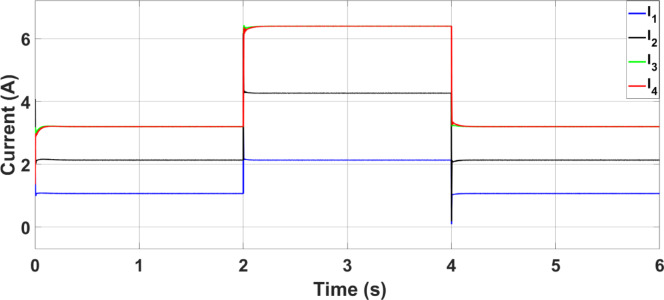
Output currents for communication delay test.

**Fig. 14:**
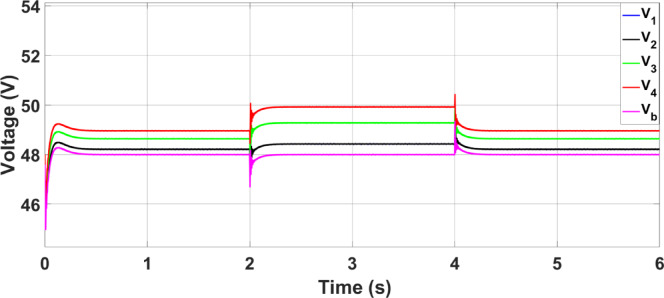
Voltages for communication delay test.

In this test, different communication delays were applied to DGUs as shown in Table 5 and sudden load changes were applied as case 1. Since there is neither queuing delay nor synchronization delay, small time delays were applied. To reduce system oscillations caused by communication delay, the cut-off frequency of voltage control loop in zero control layer for each converter was reduced by dividing each proportional gain by 5 and each integral gain by 25. Also, each converter capacitance was doubled. System response is shown in Fig. 13 and Fig. 14. Accurate current sharing was achieved despite different time delays. The maximum undershoot in load bus voltage was $2.76\%$, the maximum overshoot was $2.82\%$ and settling time was $350 \mathrm{m}\mathrm{s}.$

**Table 5:** Communication delays.

	DGU1	DGU2	DGU3	DGU4
Communication delay (ms)	$0.5$	$0.53$	$0.57$	$0.6$

## Conclusion

The paper presents a control strategy that achieves both voltage regulation and load sharing simultaneously in DC microgrids. Load sharing control prevents overloading a DGU that may trigger protection devices to disconnect it. Load sharing control can be used to achieve economic operation with minimum cost. It is also used to balance state of charge of multiple battery energy storage systems and maximize their lifetime. These applications need an adjustable load sharing ratio. A (THRO) Algorithm was used to optimize the gains of the PI controllers used. The results show that the proposed control strategy managed to achieve proper voltage regulation and load current sharing with zero steady state error under different case studies and reduced system settling time by $87.5\%$. It also reduced communication energy loss and cost. Moreover, plug and play capability was tested along with constant power load. For future work, using this control strategy with controllable load sharing ratio and bidirectional converters.

## Data Availability

The datasets used and/or analyzed during the current study are available from the corresponding author on reasonable request.
